# Influence of luminescent graphene quantum dots on trypsin activity

**DOI:** 10.2147/IJN.S155021

**Published:** 2018-03-15

**Authors:** Tanveer A Tabish, Md Zahidul I Pranjol, Ilayda Karadag, David W Horsell, Jacqueline L Whatmore, Shaowei Zhang

**Affiliations:** 1 College of Engineering, Mathematics and Physical Sciences, University of Exeter, Exeter, UK; 2 Institute of Biomedical and Clinical Science, University of Exeter Medical School, Exeter, UK

**Keywords:** graphene, enzyme, luminescence, bioavailability, surface energy

## Abstract

**Background:**

Protein–graphene interactions have the potential to play a pivotal role in the future directions of nanomedicine. These interactions lead to diverse processes such as generation of protein coronas, nano–bio interfaces, particle wrapping, and biocatalytic processes that could determine the ultimate fate of graphene nanocomposites in biologic systems. However, such interactions and their effects on the bioavailability of graphene have not yet been widely appreciated, despite the fact that this is the primary surface in contact with cells.

**Methods:**

This paper reports on the integrative physiochemical interaction between trypsin and graphene quantum dots (GQDs) to determine their potential biologic identity in enzyme engineering. This interaction was measured by a wide range of analytical methods.

**Results:**

Definitive binding and modulation of trypsin–GQDs was demonstrated for the first time by use of vibrational spectroscopy and wetting transparency, which revealed that trypsin was absorbed on GQDs’ surface through its cationic and hydrophilic residues. Our findings suggested that trypsin’s active sites were stabilized and protected by the GQDs, which were likely to be responsible for the high bioavailability of GQDs in enzymes.

**Conclusion:**

Our work demonstrates the efficacy of GQDs as an enzyme modulator with high specificity, and their great application potential in enzyme engineering as well as enzyme-based therapies.

## Introduction

The regulation of enzyme activity plays diverse roles in catalytic activity adjustments and modulation of cellular events such as signal transduction, DNA replication, metabolism, gene expression, immune responses, metastasis, and metabolism.^1^,^2^ Various types of enzyme dysfunction cause a wide variety of human diseases and disorders associated with inborn errors of metabolism and specific mutations within the enzymes.^3^–^5^ The regulation of enzyme function provides a promising direction for the development of therapeutic interventions.^6^ Hence, regulation of enzyme activity and stability have attracted a great deal of attention. Recently, luminescent quantum dots (QDs) have emerged as a promising system for enzyme modulation and regulation.^7^ These QDs have several advantages over conventional regulators: for instance, they can enter cells easily and have unique luminescent features, surface charge, hydrophilicity, and geometry and surface properties for the binding of enzymes.^8^,^9^ Recent developments in graphene nanocomposites indicate promising new pathways to control the binding and activation of protein structure and cell behavior.^10^ Several derivatives of graphene, such as graphene oxide, reduced graphene oxide, and pristine graphene, have been reported to show their interactions and influences on enzyme activities.^10^–^12^ In the past few years, graphene oxide with different functionalization and modifications has been extensively investigated to understand its interaction with proteins.^10^–^15^ The electrostatic bonding and π-π stacking interactions and covalent/noncovalent bonding are considered to be the major mechanisms of graphene–protein interactions. Graphene–biomolecule interactions have been shown to underpin clinical diagnostic tools for cancer biomarker detection, which demonstrate that graphene-based enzyme modulators are becoming an increasingly relevant alternative to traditional techniques.^10^

Graphene quantum dots (GQDs) have widely been explored in biologic applications but their interaction with enzymes has not. They are photoluminescent nanoparticles with excellent optical characteristics, unique physiochemical properties, excellent photostability, and minimal toxicity.^16^,^17^ These characteristic features make them an ideal system for biomedical applications, including drug delivery systems, diagnosis and therapy, and bioimaging and sensing.^18^ Their interactions with biomolecules form the basis of a variety of clinical and real-world applications. For this field to evolve, we need to understand the dynamic forces, surface chemistry, and the biophysiochemical nature of both components that shape these interactions. Chemical or electrostatic attachment of enzymes to GQDs could enhance the rate of nano–bio interface formation and/or cause an enzyme to denature. GQD-induced changes in biomolecular behavior and morphology would help us to better understand the bioavailability and implications of GQDs on human health and the environment.

As a biologically relevant target enzyme we selected trypsin, which is a pancreatic serine protease involved in the digestive systems of food proteins and number of important biologic activities. Trypsin is a medium-sized globular protein with applications in, for example, wound healing machineries, in washing agents involved in many biotechnology activities. The bonding forms a nano–bio interface that defines the role of the QD and can induce damage in the interacting trypsin. Features of the QD that contribute to the formation of the interface in a biologic environment are surface charge, electronic states, size, shape, functional groups, free radicals, surface roughness, and wetting properties. Features of trypsin that may influence its interaction with the QD are size, ionic strength, temperature, surface hydrophobicity, surface charge, sequence, and conformation. The trypsin–QD interactive profile may lead to dynamic changes in the living system. The interface can form when trypsin moves toward QDs. As a result, QDs can also induce potential changes to trypsin such as function and conformation as a result of surface energy release. We define how the interaction modifies the nano–bio interface and probe the trypsin activity over a range of GQD concentrations (25, 50, 75, 100, 125, and 150 µg/mL). The changes in surface and physiochemical properties as a result of enzymatic interaction of graphene are also unknown. Therefore, we utilized Raman spectroscopy, Fourier-transform infrared spectroscopy (FTIR), and wettability tests to investigate the chemical, structural, and surface hydrophilicity/hydrophobicity changes encountered by GQDs toward the stability of trypsin. Different levels of inherent surface oxygen containing functional groups of GQDs were found to be the reason behind the tuning of trypsin’s specific activity. A fluorogenic substrate for trypsin was used to carry out control experiments of trypsin activity.

## Materials and methods

### Synthesis and basic characterization of GQDs

GQDs were prepared by tuning the carbonization degree of citric acid (CA) as previously reported.^19^ In a typical procedure, 2 g CA was put into a 5 mL beaker and heated to 200°C using a heating mantle. About 5 min later, the CA was liquated. Subsequently, the color of the liquid changed from colorless to pale yellow, and then orange in 30 min, implying the formation of GQDs. The resultant orange liquid was added dropwise into 100 mL of 10 mg/mL NaOH solution, under vigorous stirring. After neutralization to pH 7.0 with NaOH, an aqueous solution of GQD was obtained.

Microstructures of as-prepared GQD samples were observed using a JOEL-2100 transmission electron microscope (TEM) at an acceleration voltage of 200 kV. Samples of GQDs were pipetted onto holey carbon Cu grids to produce the TEM specimens. Raman spectra of samples were recorded in the backscattering arrangement, using a 532 nm laser excitation at 6 mW power. FTIR spectrum of sample was recorded in the wavenumber range of 4,000–500 cm^−1^ using a Bruker Optics Tensor-27 FTIR spectrometer. The samples were mixed with KBr of spectroscopic grade and pressed to form pellets of about 5 mm in diameter and 2 mm in thickness. A Jenway 6715 UV/Vis spectrophotometer was used to collect the absorbance spectra. The photoluminescence (PL) features were obtained by using an Edinburgh Instruments Spectrofluorometer FS5 at 350 nm of excitation wavelength. The wettability of GQDs was determined using a contact angle goniometer. A digital camera was used to record the images and the contact angle was calculated (using PolyPro). The surface of the sample was prepared for wetting by gently drop casting it onto a glass slide. The surface energy was determined by measuring the contact angle of a 10 µL drop of diiodomethane (DIIO) on the surface. The equations used in the surface energy calculations are given in the “Supplementary materials”.

### Trypsin proteolytic activity on substrates and GQDs

A fluorogenic substrate, Boc-Gln-Ala-Arg-AMC (*k*_cat_/*K*_m_=2.0×10^7^ M^−1^ sec^−1^; *K*_m_=6.0 µM), at different concentrations (0, 0.1, 0.25, 0.5, and 1 µM) was used to examine trypsin-mediated enzymatic activity at 37°C at various time points (2, 5, 10, 15, 30, and 60 min). The trypsin–EDTA solution (trypsin–ethylenediaminetetraacetic acid solution 1×) was purchased from Sigma-Aldrich, Dorset, UK, and used without further purifications. The substrate stock solution was prepared in dimethyl sulfoxide and was further diluted. The test wells within a black opaque 96-well plate (Greiner Bio-one) contained 1% trypsin and various concentrations of the substrate: controls were 1% (V/V) trypsin+distilled water and substrate (v/v) only in distilled water. Plates were read at the aforementioned time points of incubation at room temperature. Plates were read at Ex/Em: 355/450 nm and the data normalized to the control (and represented as a percentage of this control). The fluorescence intensity of the substrate hydrolysis was detected kinetically using a SpectraMax plate reader. The same procedure was repeated (*n*=4) with GQDs at various concentrations (0, 25, 50, 75, 100, 125, and 150 µg/mL). The control wells contained GQDs only (dispersed in distilled water). Statistical analysis was performed between the concentration of GQDs/substrate and trypsin by unpaired Student’s *t*-test (using GraphPad Prism). Results were presented as mean ± SD, unless otherwise indicated. Values of *p*<0.05 were considered significant. FTIR, Raman spectroscopy, water contact angle (WCA), and DIIO contact angles were measured in the similar way as described in the “Synthesis and basic characterization of GQDs” section

## Results and discussion

### Basic characterization

**Figure 1:**
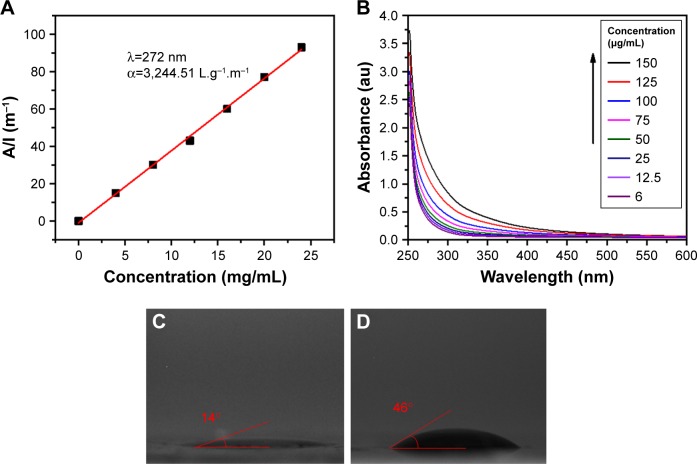
Water solubility, wetting transparency, and surface energy of GQDs. **Notes:** (**A**) The absorbance (λex =275 nm) as a function of concentration, where A and l represent absorbency and cell length respectively. The experimental data (symbols) are well described by the Lambert-Beer law (line), which indicates good water solubility of the prepared GQDs. (**B**) UV/Vis absorption spectra of GQD having concentrations of 150, 125, 100, 75, 50, 25, 12.5, and 6 µg/mL indicate band around 260 nm. (**C**) Photograph of a 10 µL drop of water on the GQDs, showing a water contact angle of 14°. (**D**) Photograph of a 10 µL drop of diiodomethane on the GQDs with a contact angle of 46°. **Abbreviation:** GQDs, graphene quantum dots.

TEM was used to observe the microstructure of GQDs (Figure S1). Dark spots shown in Figure S1A were GQDs, which had regular diameter, circular shape, and were not aggregated. TEM image shows a relatively identical size distribution between 5 and 10 nm. As shown in Figure S1B, absorption peaks centered at 1,637 and 3,402 cm^−1^ that revealed C=C and O−H bonding appeared in the FTIR spectrum. The absorptions at 1,255 and 1,078 cm^−1^ indicated the existence of C−H and C−O, respectively. Furthermore, the GQDs exhibited stretching vibrations of C−H at 2,950 and <1,350 cm^−1^, suggesting that the GQDs contained some partially carbonized CA.^20^ As shown in Figure S1C, the Raman spectrum of GQDs exhibited a D band at 1,355 cm^−1^ and a G band at 1,580 cm^−1^, which are related to a series of structure defects and the in-plane bond-stretching motion of the pairs of sp^2^ atoms, respectively.^21^ PL spectra of GQDs was almost excitation-independent, with the maximum excitation and emission wavelengths at 365 and 455 nm, respectively (Figure S1D). PL spectra of GQDs at the excitation wavelengths of 340, 350, 360, 370, and 380 nm are shown in Figure S2. Figure 1 shows that the GQDs had good water solubility (Figure 1A), and droplets of water on the surface (Figure 1C) exhibited a typical WCA of 14° indicating a strongly hydrophilic nature. The water wettability data were combined with wettability measurements of DIIO (Figure 1D) to determine the surface energy (Supplementary materials). A dispersive surface energy of 36.5 mN/m and polar surface energy of 35.7 mN/m led to a total surface energy of 72.2 mN/m.

### Trypsin activity with substrate and GQDs

**Figure 2:**
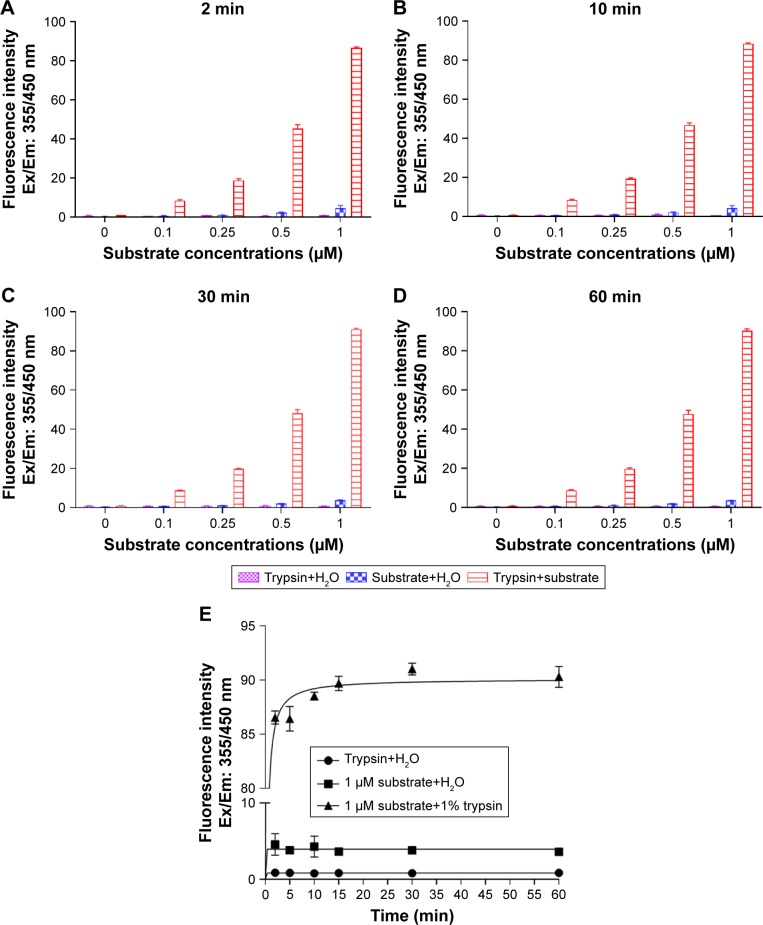
Fluorescence intensity of trypsin, substrate, and trypsin+substrate as a function of time and substrate concentration. **Notes:** Fluorogenic substrate, Boc-Gln-Ala-Arg-AMC, at different concentrations (0, 0.1, 0.25, 0.5, and 1 µm) was incubated with 1% trypsin in 96-well plates at different time points (2, 10, 30, and 60 min). (**A**–**D**) Different concentrations of substrate over different time points compared to only trypsin and substrate. (**E**) Highest concentration of substrate compared to substrate and trypsin only. Fluorescence signals were measured using plate reader at Ex/Em: 355/450 nm, where Ex and Em represents excitation and emission wavelengths. Control wells contained H_2_O+substrate and H_2_O+trypsin.

Fluorogenic substrate concentration and trypsin activity assays were conducted in order to determine the substrate breakdown and activity. Figure 2 shows that the highest concentration of substrate (1 µM) had the highest enzyme activity. In trypsin–substrate interaction, highest concentration of substrate was also active over different time points (Figure 2E). Figure 2E shows the increase in enzymatic activity over the varying concentrations of substrate.

**Figure 3:**
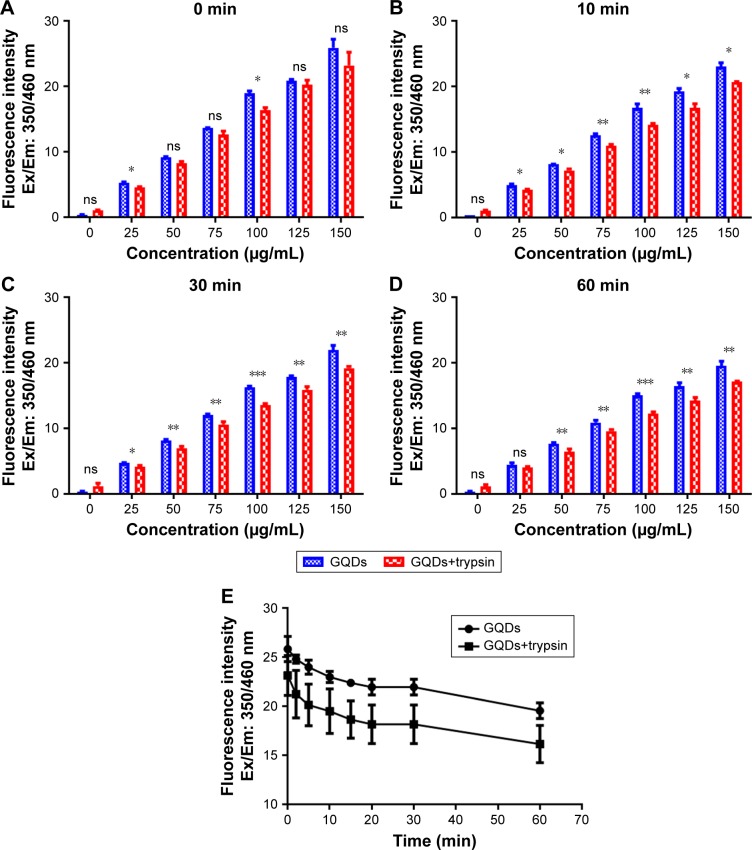
Effect of different concentrations of GQDs on trypsin activity. **Notes:** GQDs at different concentrations (150, 125, 100, 75, 50, and 25 µg/mL) were incubated with 1% trypsin in 96-well plates at different time points (2, 5, 10, 15, 30, and 60 min) as shown. (**A**–**D**) Comparison of different concentrations of GQDs on trypsin activity over 0–60 min. (**E**) Influence of the highest concentration of GQDs on trypsin activity compared to the case of GQDs only. Trypsin was highly active at 150 µg/mL concentration of GQDs and slightly active at other concentrations. Fluorescence signals were determined using plate reader at Ex/Em: 355/460 nm. *p<0.05, **p<0.01 and ***p<0.001 GQDs vs GQDs+trypsin. Control wells contained H_2_O and GQDs, and H_2_O and trypsin. n.s. denotes not significant. **Abbreviation:** GQDs, graphene quantum dots.

Figure 3 shows normalized fluorescence intensities at different concentrations of GQDs (25, 50, 75, 100, 125, and 150 µg/mL) exposed to trypsin over different time scales (0–60 min). Trypsin was active at all the concentrations of GQDs but most active at 150 µg/mL. As the concentration was decreased from 150 to 25 µg/mL, the fluorescence signals reduced. This could suggest that the trypsin was adsorbed onto the surface of GQDs via physiochemical interaction and hence block the emission of fluorescence signals from the GQDs. Decreased fluorescence intensity is also relevant to increased trypsin quenching. This may be due to the fact that water molecules are surrounded between the enzyme and the hydrophilic GQDs surface, and hence, the adsorption-induced conformational reshuffles result in revealing trypsin to water molecules. Trypsin bonding speeded up with increasing the concentration of GQDs. This behavior could indicate that both the trypsin and GQDs surface had to adapt their structures to form a stable interface. At high enzyme coverage of the GQDs surface, one could also envisage that rearrangements of protein molecules already bonded to the GQDs were required to make room for an incoming protein molecule. This crowding effect would contribute significantly to the self-fluorescence properties of GQDs.

**Figure 4:**
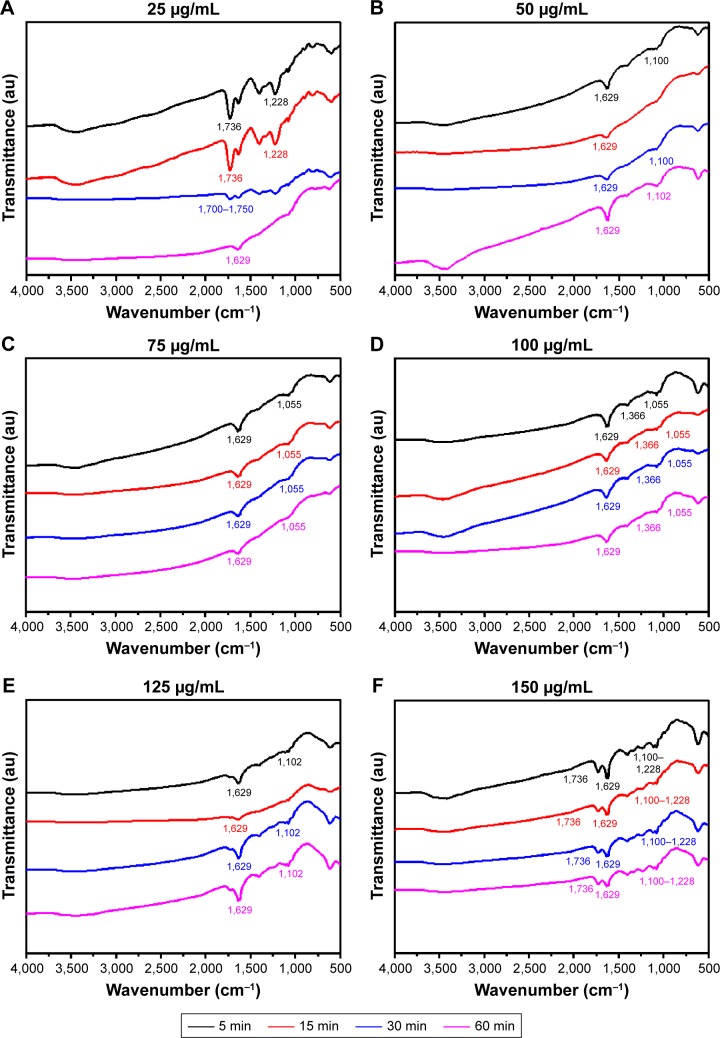
FTIR spectra of trypsin-linked GQDs. **Note:** (**A**) 25, (**B**) 50, (**C**) 75, (**D**) 100, (**E**) 125, and (**F**) 150 µg/mL GQDs concentration. **Abbreviations:** FTIR, Fourier-transform infrared spectroscopy; GQDs, graphene quantum dots.

The nano–bio interface resulting from the trypsin–GQDs interaction can be confirmed by FTIR. The changes/shifts in the functional groups of interfaces were identified by using FTIR. Figure 4A–F shows FTIR spectra of GQDs linked to trypsin at concentrations of 25, 50, 75, 10, 125, and 150 µg/mL of GQDs. The FTIR spectrum of 1% trypsin is given in Figure S3. The FTIR spectra of trypsin–GQDs interfaces exhibited a variety of trypsin absorption features such as C=O (υ_C=O_ at 1,639 cm^−1^). In particular, the C−N stretching mode peak in 100 µg/mL concentration trypsin-linked GQDs appeared at 1,366 cm^−1^ (υ_C−N_ receptor binding with an aromatic compound).^22^ The spectra of trypsin after interaction with 50 µg/mL GQDs (Figure 4B) showed not only the characteristic peaks of C=N at 1,629 cm^−1^, which arose from the amino groups of trypsin and the aldehyde groups of GQDs, but also the characteristic bands of the GQDs, 1,255 and 1,637 cm^−1^ (C−N, stretching vibration), and 1,078 cm^−1^ (C−O−C, antisymmetric vibrations) (Figure 4C). The peaks at 1,102 cm^−1^ assigned to the stretching vibration of O−H and C−O−C confirmed the presence of GQDs. Furthermore, the peak that appeared at 1,736 cm^−1^ (150 µg/mL, the highest concentration of GQDs) can be assigned to C=O, which did not appear at other concentrations except 25 µg/mL. These spectra also showed the presence of C=O (υ_C=O_ at 1,736 cm^−1^), C=C (υ_C=C_ at 1,629 cm^−1^), and at 1,228/1,055 cm^−1^ in carboxyl, epoxy, and alkoxy groups, respectively (Figure 4F). These results confirmed that trypsin had been successfully covalently bonded onto the surface of GQDs.

**Figure 5:**
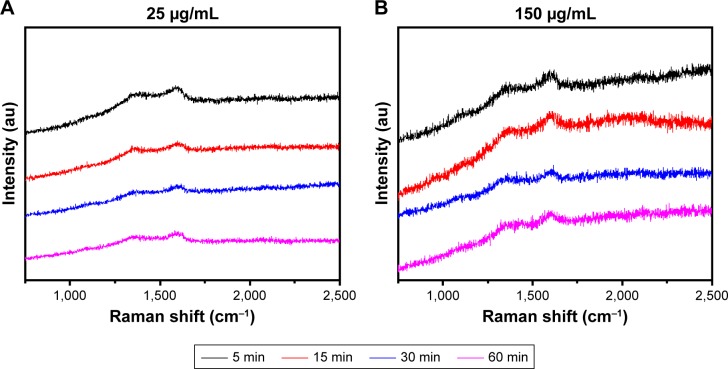
Raman spectra of trypsin-linked GQDs. **Note:** (**A**) 25 and (**B**) 150 µg/mL. **Abbreviation:** GQDs, graphene quantum dots.

Figure 5 shows Raman spectra of trypsin-linked GQDs. In the spectra of 25 and 150 µg/mL concentrations of GQDs, the amide-I vibration at 1,625 cm^−1^ arose mainly from the υ_C=O_ stretching vibration. The band in the range of 1,250–1,340 cm^−1^ was caused by the C−H_3_ and C−H_2_ deformation vibrations from the side chains of different amino acids. The amide-III was the combination of the N−H bending and C−C stretching vibration in the region 1,200–1,340 cm^−1^.^23^,^24^ Slight shifts can be observed between the two Raman spectra of GQDs and trypsin adsorbed on GQDs. In the spectrum of GQDs (Figure S1B), there were two typical peaks that appeared at ca. 1,355 and 1,580 cm^−1^. The bands at 1,600–1,625 and 1,250–1,340 cm^−1^ can be assigned to the C=O stretching of carboxylate and C−H_2_ deformation vibration. After combining with GQDs, the strong amide band at 1,629 cm^−1^ in the FTIR spectrum of trypsin appeared and merged with the band of GQDs at 1,637 cm^−1^ (C=C group). Additionally, in the Raman spectra of GQDs and trypsin–GQDs, the prominent amide band at 1,580 cm^−1^ of GQDs was shifted to 1,625 cm^−1^ in trypsin–GQDs interface. Based on these facts, it could be inferred that the trypsin interacted with GQDs through its amide bonds. However, the amide bonds might not be the only force that bonded trypsin to GQDs. Trypsin has a deep bonding pocket with an aspartic acid at the bottom. This provides the space and electrostatic complementarity to specifically bond long basic side chains, such as lysine and arginine. These are positively charged amino acids and, therefore, could be conjugated to the negatively charged surface of the GQDs through the electrostatic interaction.

**Figure 6:**
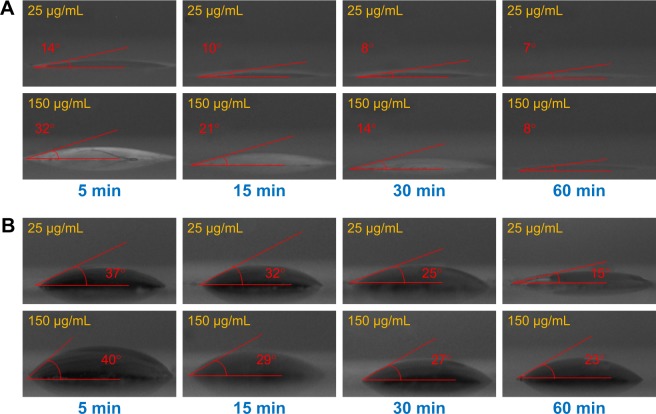
Contact angle profiles of trypsin–GQDs interfaces at 25 and 150 µg/mL concentrations of GQDs. **Notes:** (**A**) Water contact angle of interface from 5 to 60 min. (**B**) DIIO contact angle of interface from 5 to 60 min. DIIO contact was measured to calculate the surface energy of trypsin, GQDs, and their interfaces. **Abbreviations:** DIIO, diiodomethane; GQD, grapheme quantum dot.

**Figure 7:**
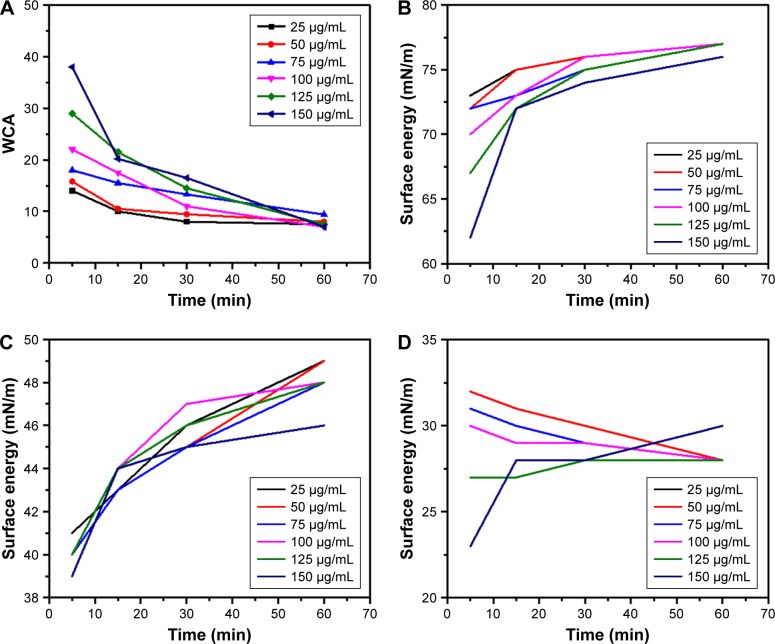
WCA and surface energy profile of GQDs–trypsin interfaces from 0 to 60 min. **Note:** (**A**) WCA, (**B**) total surface energy, (**C**) dispersive surface energy, and (**D**) polar surface energy of 25, 50, 75, 100, 125, and 150 µg/mL concentrations of GQDs treated with trypsin. **Abbreviations:** GQDs, graphene quantum dots; WCA, water contact angle.

The functional groups of GQDs act as a passivating layer and contribute to the increased hydrophilicity. To evaluate the extent of surface modification induced by trypsin, WCA measurements were carried out on the samples before and after treatment and also at different time points of trypsin–GQDs interaction (Figure 6). The trypsin displayed higher hydrolytic activity toward GQDs, as demonstrated by the decrease in the WCA values. The decrease in WCA confirmed that the reaction proceeded effectively. Upon trypsin interaction, the WCA of GQDs was moved to lower values of CAs, which indicates an increase in the surface hydrophilicity (Figure 6A). This effect was distinct and noticeable in the case of the higher concentrations, for which the average WCA value was decreased by about 30°. A decrease of 6.5° was recorded at 25 µg/mL. The decrease in DIIO contact angle (Figure 6B) revealed the surface energy profile, which is quantitatively shown in Figure 7. Overall, the results addressed a couple of key features related to the surface interaction of GQD substrates with trypsin: 1) the effect of the functional groups existing on the surface of GQDs and trypsin; 2) hydrophobicity driven by the adsorption of trypsin onto the GQDs surface to form a nano–bio interface (WCA of trypsin is shown in Figure S4). Furthermore, the rise in total and dispersive surface energy caused by the trypsin–GQDs interaction revealed that differences in functional group content, conformational flexibility, and shape and distinct bonding affinities released higher free surface energy. Higher concentrations of GQDs readily covered the surface of the trypsin to initiate the formation of a protein “soft” corona, while lower concentrations with lower yield of functional changes took over to form a corona. Polar part of total surface energy enhanced dispersion of liquid on the surface, while the dispersion section improved the hydrophobic nature and consequently increased the CA profile (Figure 7). Low polar part (Figure 7C) and high dispersion part (Figure 7D) of surface energy exhibiting different trends were evident because of the polar and nonpolar side-chains of trypsin facilitating conformational changes in the trypsin structure and consequently leading to high adsorption capacity of trypsin into GQDs. A recent study conducted by Gupta et al^25^ showed the similar surface energy profile for carbon nanotubes.

The entrapment of enzyme immobilization is generally carried out by ionic/covalent interaction, encapsulation, and adsorption. The process of adsorption is considered to be a simple, effective, and economical method for enzyme immobilization. Enzyme interactions with nanoparticle surfaces occur upon adsorption,^24^ and the adsorbed enzyme molecules in facilitating these interactions display the structure of the nanoparticle–enzyme interface. However, a key challenge in understanding the enzyme–nanoparticle interaction is to characterize the nano–bio interfaces to analyze their bulk properties such as release of surface energy, functional changes in enzyme conformation, nature of bonding, and change in wettability. The turnover product of interfacial homogeneity comes from the transfer, localization, and distribution of protein amide groups toward nanoparticles. In this regard, vibrational spectroscopic analytical methods can define the undergoing continuous changes as a result of bonding and interaction. The increased enzymatic activity of trypsin adsorbed on GQDs surface is ascribed to a definite adsorption conformation/arrangement where trypsin was adsorbed with its active site toward the surface of GQDs.

The changes identified by analytical methods in this study revealed the biosafety of GQDs. GQDs are biocompatible and friendly and more likely not to induce oxidative damage. The interaction between GQDs and trypsin is very important to reveal the influence of GQDs on enzyme activity. Vibrational spectroscopic methods and wetting transparencies have been utilized to characterize possible bonding between GQDs and trypsin. Electrostatic weak interactions may contribute to their interaction, and these weak interactions may change the conformation of trypsin, which makes its activity decreased. This work highlighted that the interactions of graphene nanocomposites with enzymes were associated with their surface chemistry. The role of tunable surface chemistry of GQDs could be exploited in the modulation and regulation of essential processes involved in cell differentiation and proliferation where trypsin plays the main role to hydrolyze proteins into smaller peptides or even amino acids. Addition of GQDs to trypsin activity could specifically and selectively favor the biocatalyst reactions, such as to improve the functional properties of trypsin such as solubility, viscosity, emulsifying features, foaming, and gelling properties, and to produce protein hydrolysates and bioactive peptides that are used in infant formulas. Immobilization of trypsin on GQDs demonstrated that GQDs are an ideal enzyme carrier. The high surface area of graphene allows significant loadings of trypsin, which results in a higher ionic strength and stability of enzymes. Further work is required to investigate the stability and thermostability of other relevant enzymes and graphene nanocomposites with specifically tailored surface properties, with the aim to further the understanding of enzyme–graphene interactions at the molecular level.

## Conclusion

We systematically studied the interactions of GQDs with trypsin to elucidate the general fate of GQDs in biological systems. GQDs exhibited a strong bonding capacity owing to their surface charge and surface functionalities. They were highly biocompatible, as demonstrated by the fact that the trypsin was adsorbed onto their surface via chemical interaction and hence blocking the emission of fluorescence signals from the graphene molecule. Furthermore, FTIR, Raman spectroscopy, and wetting transparencies of GQDs–trypsin interfaces were performed to understand the role of surface chemistry in the enzyme–GQD interactions. Detailed investigation illustrated that the GQD-induced acceleration was concentration-dependent. The results indicated that GQDs are a potential substrate for efficient enzyme immobilization. The nano–bio interface between adsorbing enzyme and GQDs surface could have potential applications in the development of biocompatible nanomaterials, nanomedicine, and for enzyme separation and purification approaches.

## Supplementary materials

### Chemical and biologic materials and reagents

All the chemicals were analytically pure and used as received. Citric acid was purchased from Alfa Aesar. Trypsin (1%), Boc-Gln-Ala-Arg-AMC fluorogenic substrate for trypsin (*k*_cat_/*K*_m_=2.0×10^7^ M^−1^ sec^−1^; *K*_m_=6.0 µM), was obtained from Enzo Life Sciences (UK) Ltd. and was stored at −20°C. Diiodomethane (product number 158429) and potassium bromide (product number P0838) were purchased from Sigma Aldrich.

### Surface energy calculations

From Young’s equation, the surface free energy of a solid (S):

$$\sigma_{S}=\sigma_{\text{SL}}+\sigma_{L}\cos\theta ,$$ (1)

where *σ*_L_ is the surface tension of the liquid (L), *σ*_SL_ is the interfacial tension between the liquid and the solid (SL), and *θ* is the contact angle formed by the liquid drop on the surface of the solid. Our aim is to determine *σ*_S_ using known *σ*_L_ and unknown *σ*_SL_. Following the Fowkes method,^1^ the interfacial tension:

$$\sigma_{\text{SL}}=\sigma_{L}+\sigma_{S}-2({\left(\sigma_{L}{\;}^{D}\sigma_{S}{\;}^{D}\right)}^{1/2}+{\left(\sigma_{L}{\;}^{P}\sigma_{S}{\;}^{P}\right)}^{1/2}),$$ (2)

where the surface energies are composed of dispersive (D) and polar (P) components. We can use this to eliminate the unknown in Equation (1).

For diiodomethane (DIIO), the liquid polar component is zero, so:

$$\sigma_{S}{\;}^{D}=\sigma_{L}{\left(\cos\theta +1\right)}^{2}/4,$$ (3)

where *σ*_L_ = *σ*_L_^D^=50.8 mN/m. From this, we directly find the dispersive component of the surface free energy of the solid from a measurement of the contact angle.

Water has both a polar and dispersive component: *σ*_L_^D^=26.4 mN/m and *σ*_L_^P^=46.4 mN/m. By rearranging equations 1 and 2, we can determine the polar component of the surface energy of the solid:

$$\sigma_{S}{\;}^{P}={\left(\sigma_{L}(\cos\theta +1)/2-{\left(\sigma_{L}{\;}^{D}\sigma_{S}{\;}^{D}\right)}^{1/2}\right)}^{2}/\sigma_{L}{\;}^{P}.$$ (4)

Once we know the dispersive and polar components, the total surface energy of the solid:

$$\sigma_{S}=\sigma_{S}{\;}^{D}+\sigma_{S}{\;}^{P}.$$

On pure samples of GQDs, DIIO formed a contact angle of 14° and water 46°. This gives surface energies of 49, 14, and 63 mN/m for the dispersive component, polar component, and total, respectively.^1^

Figure S1 Basic characterization of GQDs.
**Notes:** (**A**) Transmission electron microscopy image of GQDs showing their regular diameter, round shape, and spatial distribution. Scale bar: 200 nm. (**B**) FTIR spectrum of the GQDs showing vibrations of different functional groups. (**C**) Raman spectrum of the GQDs showing the D (1,355 cm^−1^) and G peaks (1,580 cm^−1^). (**D**) PL spectrum of the GQDs.
**Abbreviations:** FTIR, Fourier-transform infrared spectroscopy; GQDs, graphene quantum dots; PL, photoluminescence.

Figure S2 Luminescence property and emission diagram of GQDs.
**Notes:** PL spectra of GQDs at the excitation wavelength of 340, 350, 360, 370, and 380 nm. The strongest PL emission occurs at 460 nm.
**Abbreviations:** GQDs, graphene quantum dots; PL, photoluminescence.

Figure S3 FTIR of 1% trypsin.
**Notes:** Showing vibrations of C=N at 1,629 cm^−1^, stretching modes of O−H and C−O−C at 1,100–1,200 cm^−1^, and stretching vibration of C−H at 3,300–3,550 cm^−1^ as previously identified in references 2 and 3.
**Abbreviation:** FTIR, Fourier-transform infrared spectroscopy.

Figure S4 Trypsin contact angle measurements with water (left, 45º) and DIIO (right, 42º).
**Abbreviation:** DIIO, diiodomethane.

References1FowkesFMAttractive forces at interfacesInd Eng Chem196456402SahaBSaikiaJDasGCorrelating enzyme density, conformation and activity on nanoparticle surfaces in highly functional bio-nanocompositesAnalyst201514025325422540710310.1039/c4an01639d3SunJXuBShiYYangLMaHLActivity and stability of trypsin immobilized onto chitosan magnetic nanoparticlesAdv Mater Sci Eng201720171457072
