# Supplementary figures and images for: Influence of luminescent graphene quantum dots on trypsin activity
Source: Int J Nanomedicine. 2018 Mar 15;13:1525–38. doi: 10.2147/IJN.S155021 (PMC5858831; doi:10.2147/IJN.S155021)

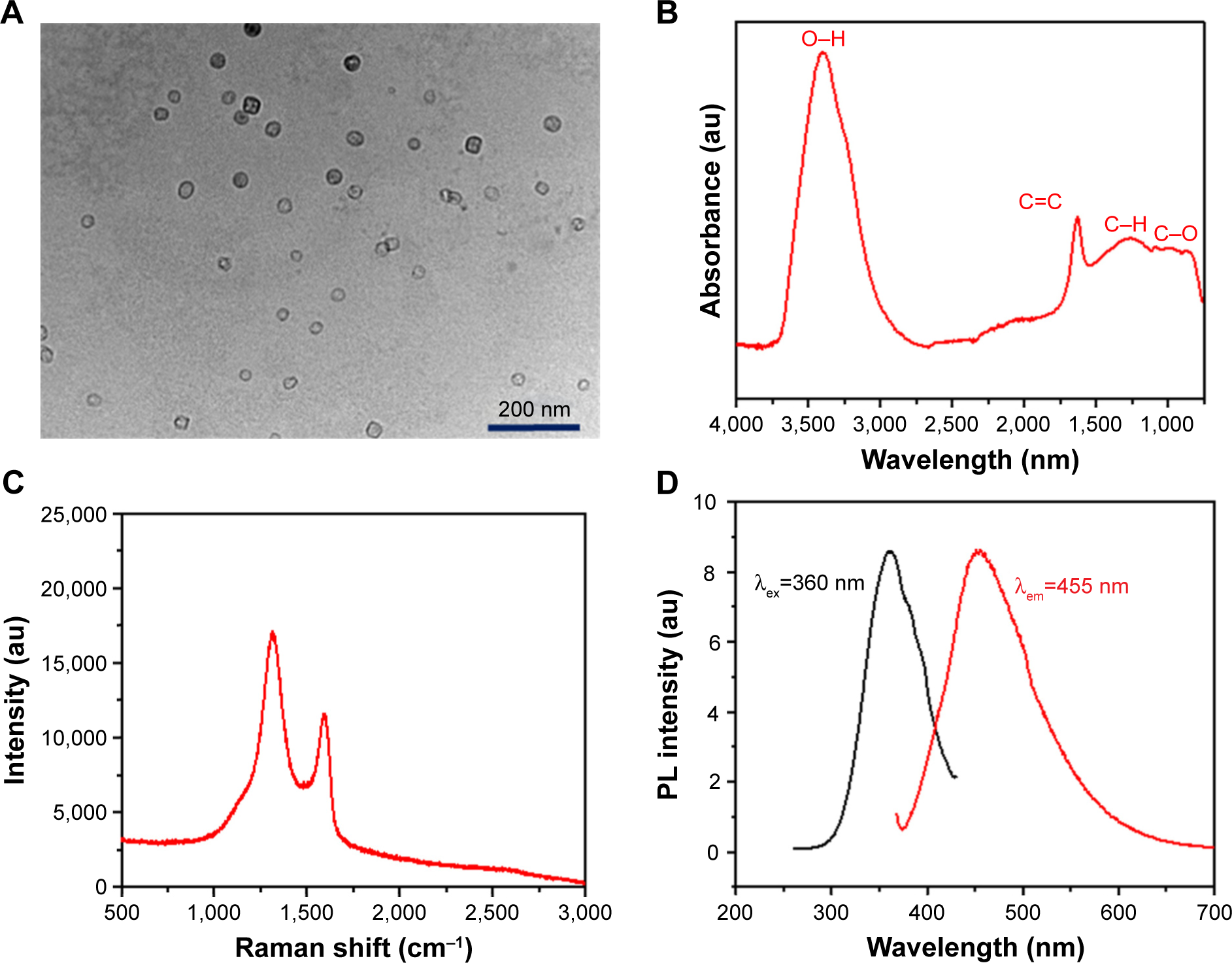

Supplement: Figure S1 — Basic characterization of GQDs. Notes: (A) Transmission electron microscopy image of GQDs showing their regular diameter, round shape, and spatial distribution. Scale bar: 200 nm. (B) FTIR spectrum of the GQDs showing vibrations of different functional groups. (C) Raman spectrum of the GQDs showing the D (1,355 cm−1) and G peaks (1,580 cm−1). (D) PL spectrum of the GQDs. Abbreviations: FTIR, Fourier-transform infrared spectroscopy; GQDs, graphene quantum dots; PL, photoluminescence. [file ijn-13-1525s1.tif]

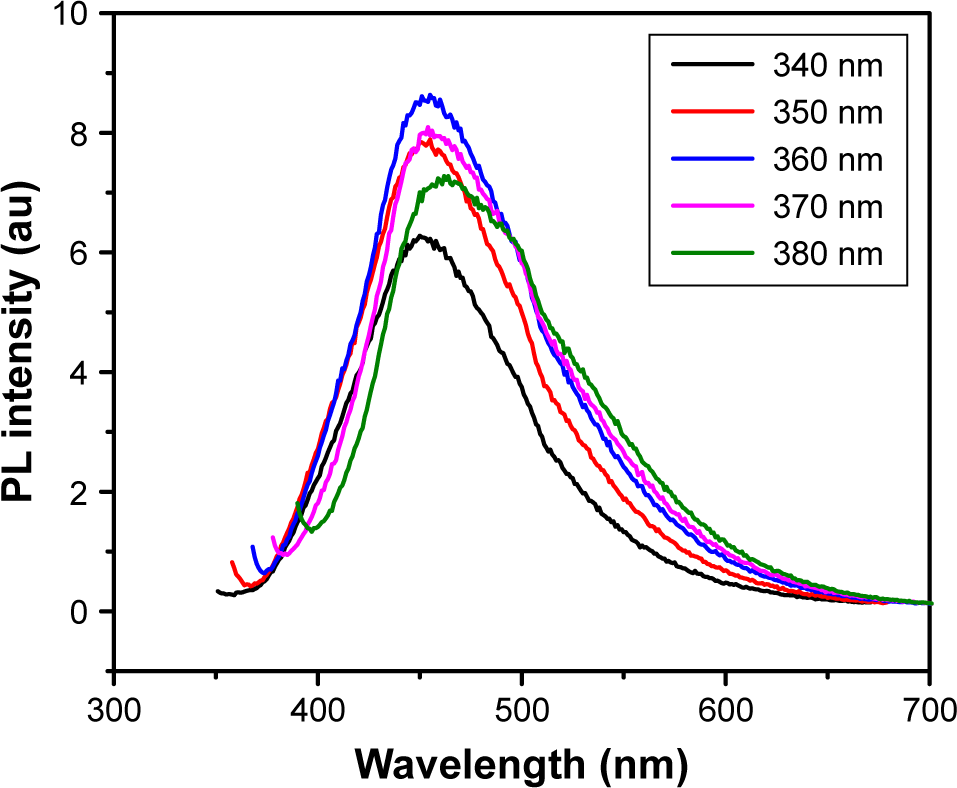

Supplement: Figure S2 — Luminescence property and emission diagram of GQDs. Notes: PL spectra of GQDs at the excitation wavelength of 340, 350, 360, 370, and 380 nm. The strongest PL emission occurs at 460 nm. Abbreviations: GQDs, graphene quantum dots; PL, photoluminescence. [file ijn-13-1525s2.tif]

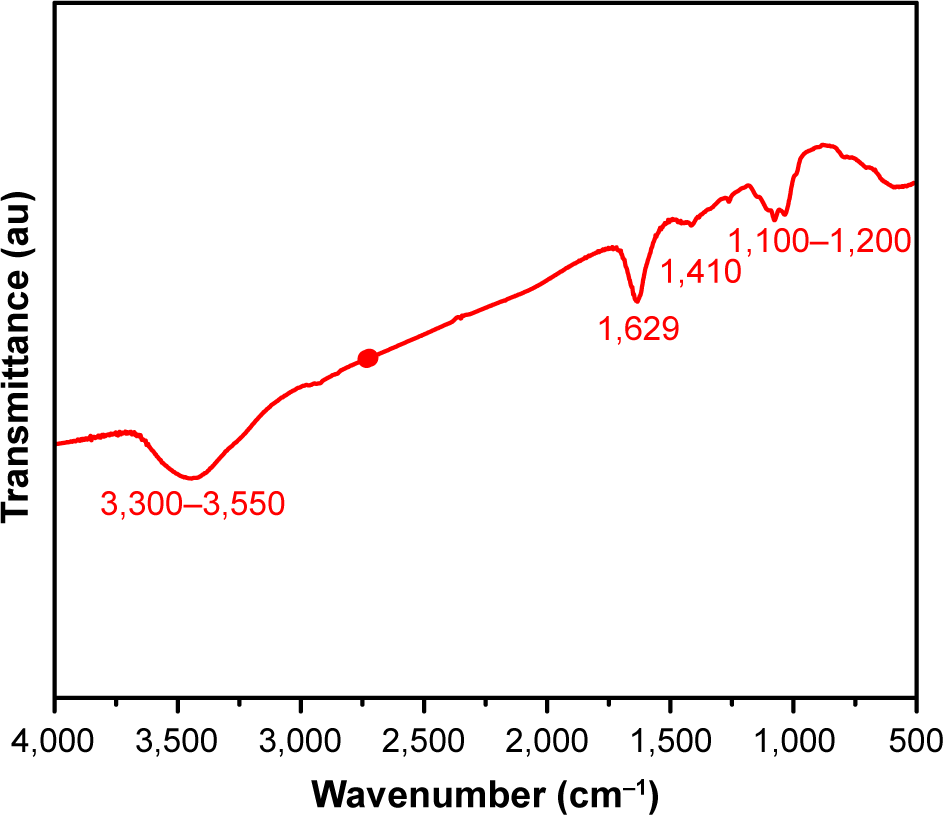

Supplement: Figure S3 — FTIR of 1% trypsin. Notes: Showing vibrations of C=N at 1,629 cm−1, stretching modes of O−H and C−O−C at 1,100–1,200 cm−1, and stretching vibration of C−H at 3,300–3,550 cm−1 as previously identified in references 2 and 3. Abbreviation: FTIR, Fourier-transform infrared spectroscopy. [file ijn-13-1525s3.tif]

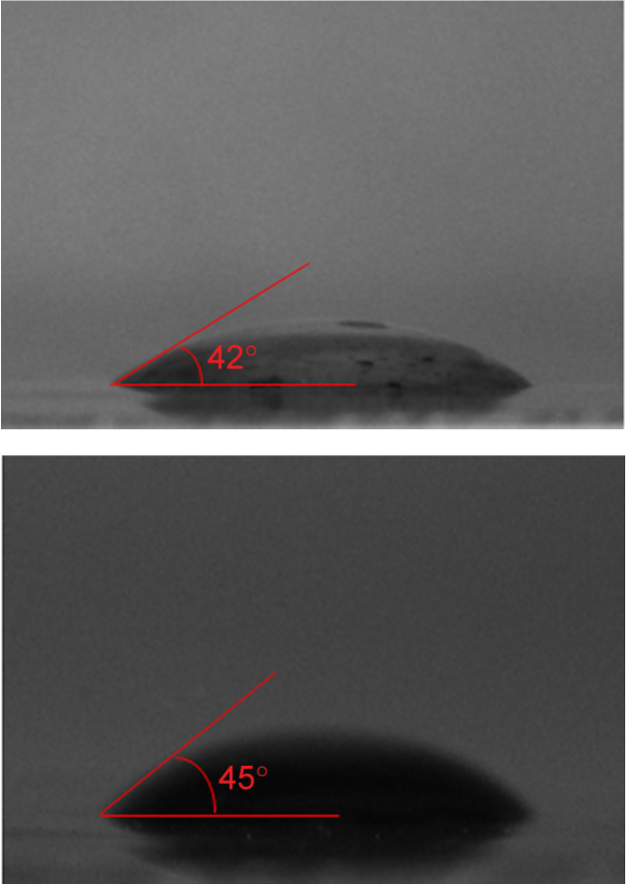

Supplement: Figure S4 — Trypsin contact angle measurements with water (left, 45º) and DIIO (right, 42º). Abbreviation: DIIO, diiodomethane. [file ijn-13-1525s4.tif]
